# Successful Identification of Nasopharyngeal Carcinoma in Nasopharyngeal Biopsies Using Deep Learning

**DOI:** 10.3390/cancers12020507

**Published:** 2020-02-22

**Authors:** Wen-Yu Chuang, Shang-Hung Chang, Wei-Hsiang Yu, Cheng-Kun Yang, Chi-Ju Yeh, Shir-Hwa Ueng, Yu-Jen Liu, Tai-Di Chen, Kuang-Hua Chen, Yi-Yin Hsieh, Yi Hsia, Tong-Hong Wang, Chuen Hsueh, Chang-Fu Kuo, Chao-Yuan Yeh

**Affiliations:** 1 Department of Pathology, Chang Gung Memorial Hospital and Chang Gung University, No. 5, Fuxing Street, Guishan District, Taoyuan City 333, Taiwan; chuang.taiwan@gmail.com (W.-Y.C.); taki.yeh@gmail.com (C.-J.Y.); susie.ueng@gmail.com (S.-H.U.); yjl777@cgmh.org.tw (Y.-J.L.); 8902028@cgmh.org.tw (T.-D.C.); sharonpooh@cgmh.org.tw (K.-H.C.); iamholy@cgmh.org.tw (Y.-Y.H.); xyz0984321@hotmail.com (Y.H.); ch9211@adm.cgmh.org.tw (C.H.); 2 Center for Vascularized Composite Allotransplantation, Chang Gung Memorial Hospital, No. 5, Fuxing Street, Guishan District, Taoyuan City 333, Taiwan; 3 Center for Big Data Analytics and Statistics, Chang Gung Memorial Hospital, No. 5, Fuxing Street, Guishan District, Taoyuan City 333, Taiwan; afen.chang@gmail.com; 4 aetherAI, Co., Ltd., No. 3-2, Yuan-Qu Street, Nangang District, Taipei City 115, Taiwan; seanyu@aetherai.com (W.-H.Y.); jimmy@aetherai.com (C.-K.Y.); 5 Chang Gung Molecular Medicine Research Center, Chang Gung University, No. 259, Wenhua First Road, Guishan District, Taoyuan City 333, Taiwan; 6 Department of Pathology, MacKay Memorial Hospital, No. 92, Section 2, Zhongshan North Road, Zhongshan District, Taipei City 104, Taiwan; 7 Tissue Bank, Chang Gung Memorial Hospital, No. 5, Fuxing Street, Guishan District, Taoyuan City 333, Taiwan; cellww@adm.cgmh.org.tw; 8 Center for Artificial Intelligence in Medicine, Chang Gung Memorial Hospital, No. 5, Fuxing Street, Guishan District, Taoyuan City 333, Taiwan; zandis@gmail.com

**Keywords:** nasopharyngeal carcinoma, deep learning, artificial intelligence, convolutional neural network, gradient-weighted class activation mapping, digital pathology, cancer identification

## Abstract

Pathologic diagnosis of nasopharyngeal carcinoma (NPC) can be challenging since most cases are nonkeratinizing carcinoma with little differentiation and many admixed lymphocytes. Our aim was to evaluate the possibility to identify NPC in nasopharyngeal biopsies using deep learning. A total of 726 nasopharyngeal biopsies were included. Among them, 100 cases were randomly selected as the testing set, 20 cases as the validation set, and all other 606 cases as the training set. All three datasets had equal numbers of NPC cases and benign cases. Manual annotation was performed. Cropped square image patches of 256 × 256 pixels were used for patch-level training, validation, and testing. The final patch-level algorithm effectively identified NPC patches, with an area under the receiver operator characteristic curve (AUC) of 0.9900. Using gradient-weighted class activation mapping, we demonstrated that the identification of NPC patches was based on morphologic features of tumor cells. At the second stage, whole-slide images were sequentially cropped into patches, inferred with the patch-level algorithm, and reconstructed into images with a smaller size for training, validation, and testing. Finally, the AUC was 0.9848 for slide-level identification of NPC. Our result shows for the first time that deep learning algorithms can identify NPC.

## 1. Introduction

Nasopharyngeal carcinoma (NPC) is a cancer with unique ethnic predisposition, Epstein–Barr virus (EBV) association, and morphologic features [1]. NPC is uncommon among Caucasians, but its incidence is disproportionally high in certain ethnic groups, including Chinese from southeastern Asia, North Africans, and the Inuit. In endemic areas, including Taiwan, most cases of NPC are of the nonkeratinizing type with EBV association [1,2,3]. Such nonkeratinizing NPC is characterized by undifferentiated or poorly differentiated carcinoma cells and a large number of admixed inflammatory cells, mainly small lymphocytes and plasma cells [1]. These morphologic features could pose difficulty in pathologic diagnosis, especially for pathologists with less experience or in nonendemic areas.

Compared to other types of medical images, the microscopic images of pathology slides are much more complicated [4]. There are multiple types of cells in a slide, and each cell type has its own morphologic characteristics, such as cell size and shape, cytoplasmic volume and features, nuclear size and shape, chromatin distribution, and number and size of nucleoli. Different cell types are then arranged in various patterns. In addition, the color and intensity of hematoxylin and eosin (H&E) staining could be influenced by subtle variations in tissue thickness and staining conditions among different laboratories, or even within a single laboratory. Furthermore, the digital files of high-resolution pathology images are extremely large in size and highly complicated, and computer analysis of such images is very difficult.

In recent years, deep neural networks have taken over as the dominant method for image recognition since their performance surpassed traditional image processing methods in the ImageNet Large Scale Visual Recognition Competition (ILSVRC) in 2012 [5]. Deep neural networks achieve superior performance through a compute-intensive learning process. Using general-purpose computing on graphics processing units (GPGPU), the deep learning process can be highly accelerated [6]. With tremendous success in recognition of natural images, convolutional neural networks have been quickly adopted for medical image analysis [7]. Therefore, analysis of pathology images using artificial intelligence (AI) is becoming more feasible recently [8,9,10,11,12,13,14,15,16,17,18,19]. Of note, a few articles described the success of AI in identifying metastatic breast cancer in lymph nodes [10,12,13,14,16,19]. The performance of AI was comparable to that of pathologists without time constraints, and adopting AI assistance in the workflow can improve the diagnostic efficiency and accuracy of pathologists.

Nasopharyngeal mucosa has abundant lymphoid cells similar to lymph nodes, but there is an additional component of benign epithelial cells. Compared to metastatic breast cancer, the much lesser differentiation of tumor cells and the large number of admixed inflammatory cells in NPC could increase the difficulty in machine identification. It would be interesting to know if AI could perform well in a morphologically challenging cancer such as NPC.

## 2. Results

### 2.1. Patch-level Model

**Figure 1:**
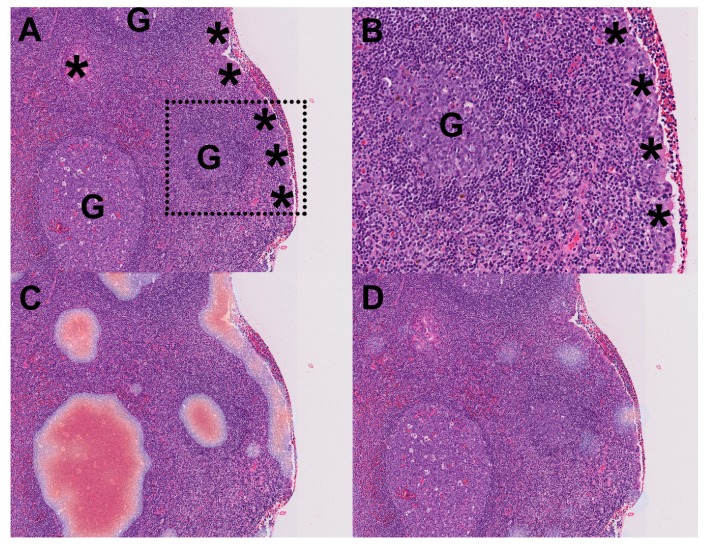
Expansion of the training data successfully prevented misclassification of patches. (**A**) An example of benign nasopharyngeal tissue in our testing set, with prominent germinal centers (G) and benign epithelial cells (asterisks) (original magnification ×40). (**B**) A magnified inset of (**A**) (dotted square). (**C**) The original patch-level model misclassified regions of germinal centers and benign epithelial cells as nasopharyngeal carcinoma (red color: high probability). (**D**) The performance of the model was markedly improved after expanding the training data with germinal centers and benign epithelial cells.

**Figure 2:**
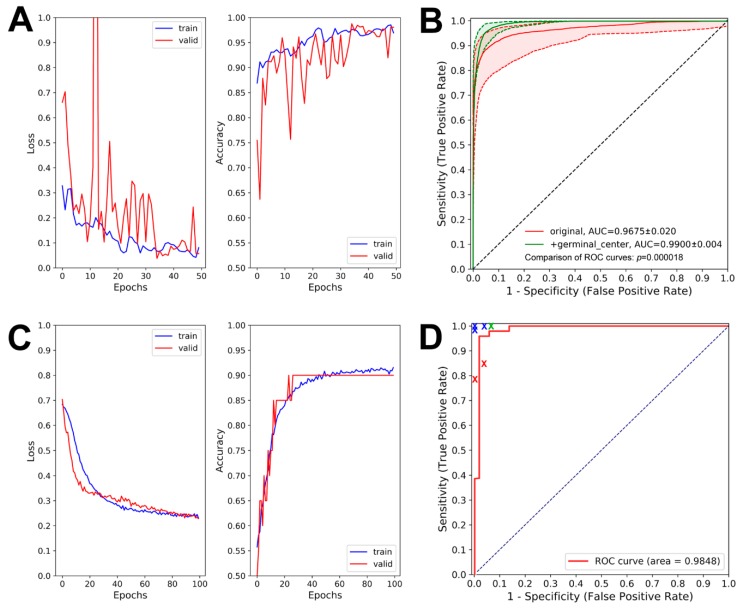
The learning curves and receiver operator characteristic (ROC) curves of our algorithms. (**A**) The learning curves of our final patch-level model. (**B**) The ROC curves of our patch-level models. The area under ROC curve (AUC) increased from 0.9675 ± 0.020 to 0.9900 ± 0.004 after expanding the training data with germinal centers and benign epithelial cells (*p* = 0.000018). (**C**) The learning curves of our slide-level model. (**D**) The ROC curve of our slide-level model. The results of pathology residents (red crosses), chief resident (green cross), and attending pathologists (blue crosses) were included for comparison.

During patch-level learning, we noticed that patches of benign nasopharynx with certain morphologic features, including germinal centers and benign epithelial cells, tended to be misclassified as NPC (Figure 1A–C). To prevent such misclassification, we added 5634 areas of benign epithelial cells and 1021 areas of germinal centers for patch-level learning. The retrained patch-level model successfully recognized germinal centers and benign epithelial cells as benign tissue (Figure 1D). The learning curves of our final patch-level model are shown in Figure 2A. The receiver operating characteristic (ROC) curves of our original and final patch-level models are shown in Figure 2B. The area under the ROC curve (AUC) showed significant increase (*p* = 0.000018) from 0.9675 ± 0.020 (original patch-level model) to 0.9900 ± 0.004 (final patch-level model).

### 2.2. Key Morphologic Features for Patch-level Identification

**Figure 3:**
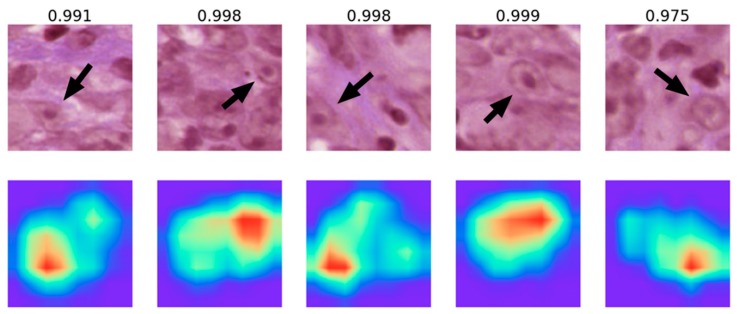
Results of gradient-weighted class activation mapping (Grad-CAM) on patches (256 × 256 pixels) classified as nasopharyngeal carcinoma (NPC) by our patch-level model. The lower row is the result of Grad-CAM, and the upper row is the corresponding hematoxylin and eosin (H&E) images for comparison. The numbers above each H&E image represent the probability of NPC produced by our patch-level algorithm. Note that the most important region (red color) for classifying a patch as NPC correlated with the location of a clearly identifiable cancer cell (arrows).

The results of gradient-weighted class activation mapping for representative NPC patches are shown in Figure 3. The relatively important regions for our final patch-level NPC identification in each patch (Figure 3; red areas) were the locations of clearly identifiable cancer cells (Figure 3; arrows). Our result confirmed that the patch-level model did identify the morphologic features of NPC tumor cells.

### 2.3. Slide-level Model

**Figure 4:**
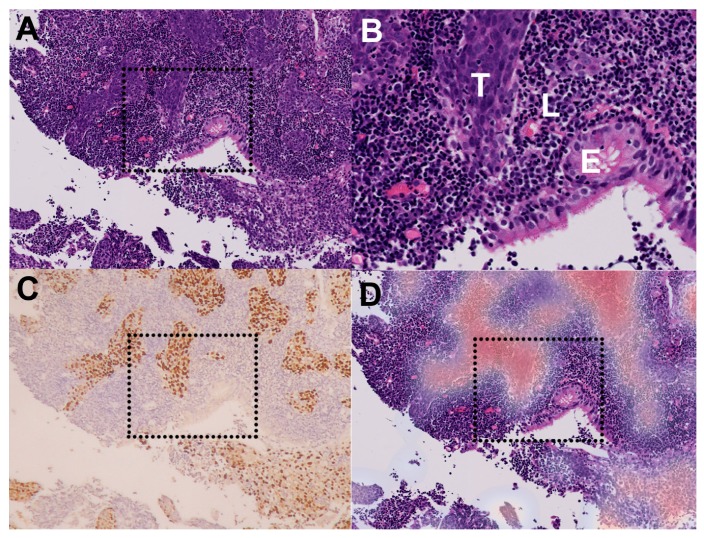
Correlation between our patch-level model and Epstein–Barr virus (EBV)-encoded small RNA (EBER) in situ hybridization. (**A**) An example of nasopharyngeal carcinoma in our testing set (original magnification ×40). (**B**) A magnified inset of (**A**) (dotted square). Note the tumor cells (T), lymphoid stroma (L), and benign epithelium (E). (**C**) The tumor cells were highlighted by EBER in situ hybridization. (**D**) The tumor areas identified by our patch-level model (red color: high probability) correlated well with (**C**).

**Figure 5:**
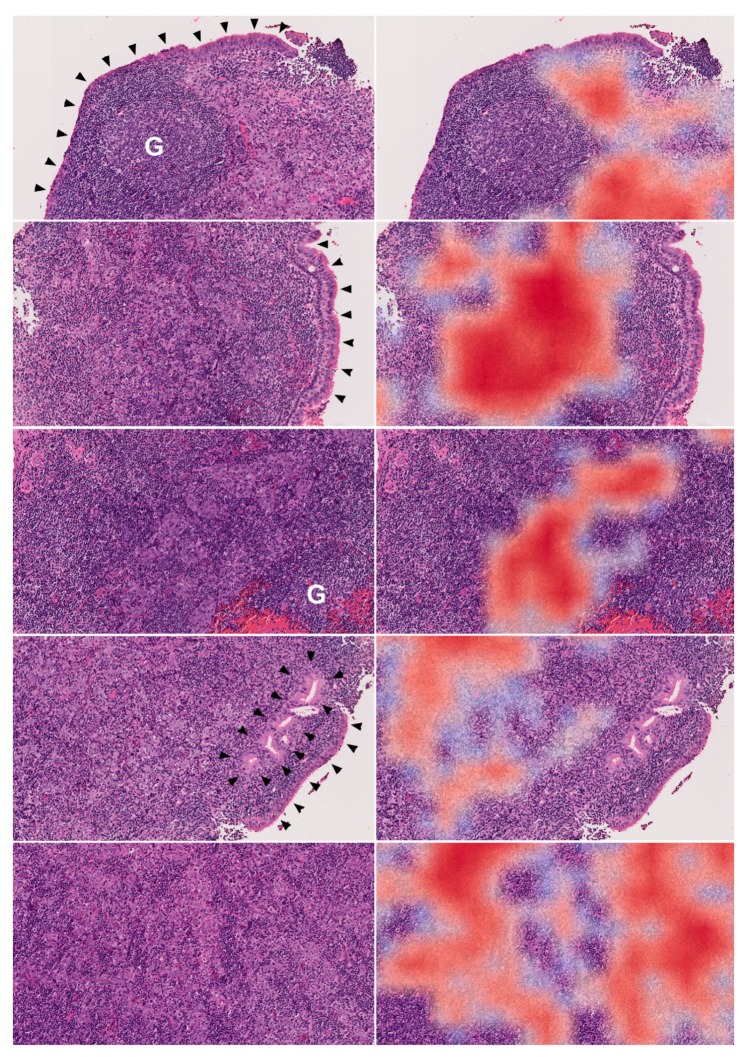
Correlation between our patch-level model and H&E morphology. More examples of nasopharyngeal carcinoma in our testing set. Each right image is derived from the left corresponding H&E image using our patch-level model (original magnification ×100). Despite the large number of admixed lymphocytes, the tumor cells were successfully identified by our patch-level mode (red color: high probability). Note that the germinal centers (G) and benign epithelial cells (arrowheads) were correctly classified as benign.

An example of our whole-slide image analyzed by our patch-level model is shown in Figure 4. Our patch-level model successfully identified areas of cancer cells (Figure 4D) with similarity to the areas identified by EBV-encoded small RNA (EBER) in situ hybridization (Figure 4C). More examples of NPC are shown in Figure 5. Despite the presence of many admixed lymphocytes (Figure 5; left panels), our patch-level model effectively identified tumor cells (Figure 5; right panels). The learning curves of our slide-level model are shown in Figure 2C. The ROC curve of our slide-level model is shown in Figure 2D. The AUC of our slide-level model was 0.9848.

The results of our human NPC identification, including sensitivity and specificity, are also shown in Figure 2D. The performance of our slide-level model was comparable to that of pathology residents (red crosses) but slightly worse than that of attending pathologists (blue crosses) and the chief resident (green cross).

## 3. Discussion

Here we show for the first time that deep convolutional neural networks can identify NPC, a cancer with little differentiation and many admixed inflammatory cells. Compared to previous studies identifying metastatic breast cancer in lymph nodes [10,12,13,14,16,19], identification of NPC with AI is certainly a more difficult task. The tumor cells of NPC are mostly undifferentiated or poorly differentiated, resulting in more morphologic similarities to germinal center cells. In addition, the admixed inflammatory cells in NPC tumor cell clusters also increase the difficulty in machine identification. Most importantly, benign epithelial cells rarely appear in axillary lymph nodes (except for rare glandular inclusions [20]), whereas most nasopharyngeal biopsies include benign epithelial cells, both on the surface and in the stroma. The distinction between malignant and benign epithelial cells is also a challenge.

Indeed, during our original patch-level learning, we found that the patch-level model tended to misclassify germinal centers and benign epithelial cells as NPC. Since only a small proportion of our original benign patches in the training set contained germinal centers and benign epithelial cells, the misclassification was likely due to under-representation of these areas in the training data. To overcome this, large numbers of areas containing germinal centers and benign epithelial cells were further annotated, and patches cropped from these areas were added into the training materials. The expansion of the training data with these patches successfully prevented the misclassification of germinal centers and benign epithelial cells as NPC (Figure 1).

Although deep neural networks can be used to identify images, it is difficult to know the exact morphologic features identified by the machine during complicated computations. Gradient-weighted class activation mapping is a useful tool to visualize the decision making in a deep neural network [21]. It is known that deeper layers of a neural network identify more complicated structures [22,23]. Utilizing the gradients flowing into the final layer, a coarse localization map highlighting the important areas for image classification can be produced. Using the gradient-weighted class activation mapping, we demonstrated that our patch-level model classifies patch images as NPC mainly according to the areas with clearly identifiable cancer cells (Figure 3).

The accuracy of our slide-level model was similar to that of our pathology residents but slightly worse than that of our attending pathologists (Figure 2D). For the few NPC cases misclassified as benign nasopharynx in our testing set, the tumor area percentage was very low (<5%). Although these cases were missed by our slide-level model, the areas of cancer cells were successfully identified by our patch-level model. The relative paucity of NPC cases with a very low tumor percentage in our training set could explain these few failed cases in our slide-level model.

The development of deep learning algorithms to identify pathology images is largely hindered by the time-consuming manual annotation. Recently, a study showed that using multiple instance learning, a clinical-grade algorithm can be produced using a large dataset of whole-slide images without manual annotation [8]. However, they used around 10,000 whole-slide images (9894 to 12,727 slides) in each dataset to develop an algorithm. For uncommon cancer types such as NPC, it is not feasible to obtain 10,000 cases for multiple instance learning. For now, high-quality manual annotation is inevitable to develop AI pathology for uncommon entities.

Since our images were manually annotated by a single senior pathologist, a bias due to personal subjectivity cannot be excluded. In addition, the color and intensity of H&E staining tend to differ among laboratories. Since our algorithms were developed using slides from a single laboratory, the accuracy could decrease for cases from other institutions. Expanding our training data with cases from other laboratories should help to achieve a more robust performance in the future.

It has been proposed that using AI as a screening tool, pathologists could exclude 65%–75% of slides but still retain 100% sensitivity [8]. It is noteworthy that AI is usually trained for a specific task, and an answer outside the training set cannot be made by AI. For example, we cannot expect our algorithms to detect the rare cases of nasopharyngeal lymphoma, which was not included in our training data. It could be a safer approach to use AI as an assisting tool to highlight tumor areas for pathologists, since we might encounter rare tumors outside training sets in daily practice.

## 4. Materials and Methods

### 4.1. Case Selection

A total of 726 nasopharyngeal biopsies (from the year 2015 to 2018), including 363 cases of NPC (354 non-keratinizing and 9 keratinizing) and 363 cases of benign nasopharyngeal tissue, were retrieved from the archives of Department of Pathology, Chang Gung Memorial Hospital in Linkou, Taiwan. For each case, one H&E slide was used. The H&E slides of all cases were reviewed by two senior pathologists (W.-Y.C. and C.H.) under a dual-head microscope to confirm the diagnosis. Whole-slide high-resolution digital images were produced using a NanoZoomer S360 digital slide scanner (Hamamatsu Photonics, Hamamatsu, Japan) with a 40× objective mode. The size of a whole-slide image was 179166 ± 25384 × 76096 ± 16180 pixels on average. We randomly selected 100 cases as the testing set, 20 cases as the validation set, and 606 other cases that were used as the training set. All three datasets had equal numbers of NPC cases and benign cases. This study had been approved by the Institutional Review Board of Chang Gung Memorial Hospital (IRB No. 201800287B0), Permission date (2 March 2018).

### 4.2. Computer Hardware and Softwares

We performed our experiments on a customized server with 1× Nvidia Tesla P40 graphics processing unit (GPU). Our algorithms were developed with Python 3.6 and TensorFlow 1.12 on a Linux platform.

### 4.3. Patch-level Model

Areas of NPC, benign nasopharynx, and background were annotated by a senior pathologist (W.-Y.C.) using a free-hand region-of-interest tool on the aetherAI digital pathology platform (aetherAI, Taipei, Taiwan). The annotation was mainly based on morphology of the H&E images. For difficult cases, the results of cytokeratin immunostaining and/or EBER in situ hybridization were used as assistance. The total numbers of annotated free-hand areas of NPC, benign tissue, and background (areas without tissue) were 6548, 10,165, and 62, respectively. For the improvement of patch-level learning, 5634 areas of benign epithelium and 1021 areas of germinal centers were subsequently annotated.

For patch-level training, validation, and testing, square image patches of 256 × 256 pixels were randomly and dynamically cropped from the free-hand annotated areas. For patches of background and benign tissue, the cropped patches must be 100% within the annotated region. For NPC patches, at least 50% of the patch area must be within the annotated tumor region. For each class (NPC, benign tissue or background), around 840,000 patches were sampled.

Our patch-level training was performed using ResNeXt, a deep neural network with a residual and inception architecture [24,25,26]. We initialized the kernel weights from existing models pretrained on another domain. Along with the training progress, the kernel weights were gradually updated with the process called stochastic gradient descent (SGD) [27], which repeated millions of times. We used the SGD optimizer with the Nesterov momentum [28] (with an initial learning rate of 0.0017 and a momentum of 0.95) to train the model and evaluated the performance per 1000 training steps. A batch size of balanced 48 patches (16 each of NPC, benign tissue, and background) was used to train the model. We used a scheme to reduce the learning rate on plateau.

To compare the ROC curves of our original and final patch-level models, we repeated the testing inference process 30 times. In each testing run, the two models predicted the same randomly taken 16,000 testing patches. The mean and variance of AUC of the two models were calculated. The performance of the two models was compared using a two-tailed *t*-test.

### 4.4. Gradient-weighted Class Activation Mapping

We used gradient-weighted class activation mapping [21] to visualize relatively important regions for model prediction in a patch. Briefly, a localization map of important regions in the patch was produced using the gradient information flowing into the final layer of the neural network [21].

### 4.5. Slide-level Model

All of our 726 whole-slide images were sequentially cropped into patches of 256 × 256 pixels without overlapping, and each patch was inferred with the patch-level algorithm. The inferred results were reconstructed into a whole-slide image of a smaller size, with each pixel containing three channels: probability of NPC, benign tissue, and background. The reconstructed images were used for training (606 images in the training set), validation (20 images in the validation set), and testing (100 images in the testing set).

Due to variation in image size among slides, we resized inputs into certain shapes per batch. The target sizes included 256 × 256, 256 × 512, 256 × 768, 512 × 768, 400 × 600, and 500 × 500 pixels. Resizing inputs into different target sizes per batch worked as data augmentation and improved the model generalization. We combined the inferred probability maps and corresponding low-resolution whole slide images and utilized a residual network ResNet [25] to train the slide-level model with free input sizes. The advantage of using free size input is that any given slide can be verified multiple times under different aspect ratios. During the training process, we used the SGD optimizer with the Nesterov momentum [28] (with an initial learning rate of 0.0017 and a momentum of 0.95) and set a balanced batch size of 8 (4 each of NPC and benign nasopharynx). A scheme to reduce the learning rate on plateau was also used to optimize the model performance.

### 4.6. Comparison with Human Performance

Four attending pathologists (C.-J.Y., S.-H.U., T.-D.C., and K.-H.C.), one chief resident (Y.-J.L.), and two residents (Y.-Y.H. and Y.H.) from the Department of Pathology, Chang Gung Memorial Hospital in Linkou participated in this comparison. The 100 H&E slides of the testing set were reviewed under a microscope by each participant without time constraints. The slides of auxiliary studies, such as immunohistochemistry and in situ hybridization, were not provided. For each participant, the total slide review process took 43 to 58 min, with a median of 54 min. The sensitivity and specificity of human reviewers were compared with those of our slide-level model produced by deep learning.

## 5. Conclusions

In the present study, we have developed for the first time deep learning algorithms to identify NPC in nasopharyngeal biopsies. Despite the lack of tumor cell differentiation and the abundance of admixed inflammatory cells in NPC, we show that deep learning can overcome these difficulties. Expansion of the training data with misclassified patches can improve the patch-level performance, and gradient-weighted class activation mapping is helpful to confirm the morphologic features used in machine identification.

## References

[1] Petersson B.F., Bell D., El-Mofty S.K., Gillison M., Lewis J.S., Nadal A., Nicolai P., Wenig B.M., El-Naggar A.K., Chan J.K.C., Grandis J.R., Takata T., Slootweg P.J. (2017). Nasopharyngeal carcinoma. WHO Classification of Head and Neck Tumours.

[2] Andersson-Anvret M., Forsby N., Klein G., Henle W. (1977). Relationship between the Epstein-Barr virus and undifferentiated nasopharyngeal carcinoma: correlated nucleic acid hybridization and histopathological examination. Int. J. Cancer.

[3] Klein G., Giovanella B.C., Lindahl T., Fialkow P.J., Singh S., Stehlin J.S. (1974). Direct evidence for the presence of Epstein-Barr virus DNA and nuclear antigen in malignant epithelial cells from patients with poorly differentiated carcinoma of the nasopharynx. Proc. Natl. Acad. Sci. USA.

[4] Gurcan M.N., Boucheron L.E., Can A., Madabhushi A., Rajpoot N.M., Yener B. (2009). Histopathological Image Analysis: A Review. IEEE Rev. Biomed. Eng..

[5] Krizhevsky A., Sutskever I., Hinton G.E., Bartlett P., Pereira F.C.N., Burges C.J.C., Bottou L., Weinberger K.Q. (2012). ImageNet Classification with Deep Convolutional Neural Networks. Advances in Neural Information Processing Systems 25.

[6] Gomez L.B., Cappello F., Carro L., DeBardeleben N., Fang B., Gurumurthi S., Pattabiraman K., Rech P., Reorda M.S. GPGPUs: How to Combine High Computational Power with High Reliability. https://ieeexplore.ieee.org/document/6800555.

[7] Litjens G., Kooi T., Bejnordi B.E., Setio A.A.A., Ciompi F., Ghafoorian M., van der Laak J., van Ginneken B., Sanchez C.I. (2017). A survey on deep learning in medical image analysis. Med. Image Anal..

[8] Campanella G., Hanna M.G., Geneslaw L., Miraflor A., Werneck Krauss Silva V., Busam K.J., Brogi E., Reuter V.E., Klimstra D.S., Fuchs T.J. (2019). Clinical-grade computational pathology using weakly supervised deep learning on whole slide images. Nat. Med..

[9] Ehteshami Bejnordi B., Mullooly M., Pfeiffer R.M., Fan S., Vacek P.M., Weaver D.L., Herschorn S., Brinton L.A., van Ginneken B., Karssemeijer N. (2018). Using deep convolutional neural networks to identify and classify tumor-associated stroma in diagnostic breast biopsies. Mod. Pathol..

[10] Holten-Rossing H., Talman M.M., Jylling A.M.B., Laenkholm A.V., Kristensson M., Vainer B. (2017). Application of automated image analysis reduces the workload of manual screening of sentinel lymph node biopsies in breast cancer. Histopathology.

[11] Korbar B., Olofson A.M., Miraflor A.P., Nicka C.M., Suriawinata M.A., Torresani L., Suriawinata A.A., Hassanpour S. (2017). Deep Learning for Classification of Colorectal Polyps on Whole-slide Images. J. Pathol. Inform..

[12] Litjens G., Sanchez C.I., Timofeeva N., Hermsen M., Nagtegaal I., Kovacs I., Hulsbergen-van de Kaa C., Bult P., van Ginneken B., van der Laak J. (2016). Deep learning as a tool for increased accuracy and efficiency of histopathological diagnosis. Sci. Rep..

[13] Liu Y., Kohlberger T., Norouzi M., Dahl G.E., Smith J.L., Mohtashamian A., Olson N., Peng L.H., Hipp J.D., Stumpe M.C. (2019). Artificial Intelligence-Based Breast Cancer Nodal Metastasis Detection: Insights Into the Black Box for Pathologists. Arch. Pathol. Lab. Med..

[14] Steiner D.F., MacDonald R., Liu Y., Truszkowski P., Hipp J.D., Gammage C., Thng F., Peng L., Stumpe M.C. (2018). Impact of Deep Learning Assistance on the Histopathologic Review of Lymph Nodes for Metastatic Breast Cancer. Am. J. Surg. Pathol..

[15] Sun M., Zhou W., Qi X., Zhang G., Girnita L., Seregard S., Grossniklaus H.E., Yao Z., Zhou X., Stalhammar G. (2019). Prediction of BAP1 Expression in Uveal Melanoma Using Densely-Connected Deep Classification Networks. Cancers.

[16] Valkonen M., Kartasalo K., Liimatainen K., Nykter M., Latonen L., Ruusuvuori P. (2017). Metastasis detection from whole slide images using local features and random forests. Cytometry A.

[17] Wang S., Yang D.M., Rong R., Zhan X., Fujimoto J., Liu H., Minna J., Wistuba I.I., Xie Y., Xiao G. (2019). Artificial Intelligence in Lung Cancer Pathology Image Analysis. Cancers.

[18] Xu J., Luo X., Wang G., Gilmore H., Madabhushi A. (2016). A Deep Convolutional Neural Network for segmenting and classifying epithelial and stromal regions in histopathological images. Neurocomputing.

[19] Ehteshami Bejnordi B., Veta M., Johannes van Diest P., van Ginneken B., Karssemeijer N., Litjens G., van der Laak J., Hermsen M., Manson Q.F., Balkenhol M. (2017). Diagnostic Assessment of Deep Learning Algorithms for Detection of Lymph Node Metastases in Women With Breast Cancer. JAMA.

[20] Fellegara G., Carcangiu M.L., Rosai J. (2011). Benign epithelial inclusions in axillary lymph nodes: report of 18 cases and review of the literature. Am. J. Surg Pathol.

[21] Selvaraju R.R., Cogswell M., Das A., Vedantam R., Parikh D., Batra D. Grad-CAM: Visual Explanations from Deep Networks via Gradient-Based Localization. https://arxiv.org/abs/1610.02391.

[22] Mahendran A., Vedaldi A. (2016). Visualizing Deep Convolutional Neural Networks Using Natural Pre-images. Int. J. Comput. Vis..

[23] Bengio Y., Courville A., Vincent P. (2013). Representation Learning: A Review and New Perspectives. IEEE Trans. Pattern Anal. Mach. Intell..

[24] Xie S., Girshick R., Dollár P., Tu Z., He K. Aggregated Residual Transformations for Deep Neural Networks. https://ieeexplore.ieee.org/document/8100117.

[25] He K., Zhang X., Ren S., Sun J. Deep Residual Learning for Image Recognition. https://ieeexplore.ieee.org/document/7780459.

[26] Szegedy C., Liu W., Jia Y., Sermanet P., Reed S., Anguelov D., Erhan D., Vanhoucke V., Rabinovich A. Going Deeper with Convolutions. https://ieeexplore.ieee.org/document/7298594.

[27] Bottou L. Large-Scale Machine Learning with Stochastic Gradient Descent. Proceedings of the Computational Statistics 2010.

[28] Sutskever I., Martens J., Dahl G., Hinton G. On the importance of initialization and momentum in deep learning. Proceedings of the 30th International Conference on Machine Learning.

